# The Diagnostic Value of the Combination of Serum Brain-Derived Neurotrophic Factor and Insulin-Like Growth Factor-1 for Major Depressive Disorder Diagnosis and Treatment Efficacy

**DOI:** 10.3389/fpsyt.2020.00800

**Published:** 2020-08-13

**Authors:** Alexandra S. Troyan, Oleg A. Levada

**Affiliations:** Psychiatry Course, State Institution “Zaporizhzhia Medical Academy of Postgraduate Education Ministry of Health of Ukraine”, Zaporizhzhia, Ukraine

**Keywords:** major depressive disorder, insulin-like growth factor-1, brain-derived neurotrophic factor, biomarkers, cognitive functions, vortioxetine

## Abstract

**Background:**

Last decades of psychiatric investigations have been marked by a search for biological markers that can clarify etiology and pathogenesis, confirm the diagnosis, screen individuals at risk, define the severity, and predict the course of mental disorders. In our study, we aimed to evaluate if BDNF and IGF-1 serum concentrations separately and in combination might be used as biomarkers for major depressive disorder (MDD) diagnosis and treatment efficacy and to evaluate the relationships among those proteins and clinical parameters of MDD.

**Methods:**

Forty-one MDD patients (according to DSM-5) and 32 healthy controls (HC) were included in this study. BDNF and IGF-1 serum concentrations, psychopathological (MADRS, CGI) and neuropsychological parameters (PDQ-5, RAVLT, TMT-B, DSST), functioning according to Sheehan Disability Scale were analyzed in all subjects at admission and 30 MDD patients after 8 weeks of vortioxetine treatment. Correlational analyses were performed to explore relationships between BDNF and IGF-1 and clinical characteristics. AUC-ROCs were calculated to determine if the value of serum BDNF and IGF-1 levels could serve for MDD diagnosis.

**Results:**

MDD patients had significantly lower serum BDNF (727.6 ± 87.9 pg/ml vs. 853.0 ± 93.9 pg/ml) and higher serum IGF-1 levels (289.15 ± 125.3 ng/ml vs. 170.2 ± 58.2 ng/ml) compared to HC. Significant correlations were obtained between BDNF levels and MDD status, depressive episode (DE) severity, precipitating factors, executive functions disruption (TMT-B, RAVLT immediate recall scores) and all subdomains of functioning. As for IGF-1, correlations were found between IGF-1 level and MDD status, DE severity, number and duration of DE, parameters of subjective and objective cognitive functioning (PDQ-5, RAVLT, TMT-B, DSST scores), and all subdomains of functioning. The associations between IGF-1 concentrations and cognitive tests’ performance were stronger than those of BDNF. Separately both BDNF and IGF-1 demonstrated good discriminating ability for MDD diagnosis with AUC of 0.840 and 0.824, respectively. However, the combination of those neurotrophins had excellent diagnostic power to discriminate MDD patients from HC, providing an AUC of 0.916. Vortioxetine treatment significantly increased BDNF and attenuated IGF-1 serum concentrations, improved all psychopathological and neuropsychological parameters and functioning.

**Conclusions:**

The combination of IGF-1 and BDNF might be considered as a diagnostic combination for MDD.

## Introduction

The last decades of psychiatric investigations have been marked by a search for biological markers that can clarify etiology and pathogenesis, confirm a diagnosis, screen individuals at risk, define the severity, and predict the course of mental disorders (1–3). With respect to major depressive disorder (MDD), abnormal neuroplasticity in cerebral regions, responsible for emotional and cognitive processing, is considered to be one of the key pathogenic mechanisms (4, 5) that potentially have a biomarker value. It is associated with alterations in the expression of neurotrophic factors, such as brain-derived neurotrophic factor (BDNF), neurotrophin-3, neurotrophin-4/5, nerve growth factor, insulin-like growth factor (IGF-1), etc. (6–8). The vital role of the neurotrophins is explained by their involvement in the processes of neuronal growth, differentiation, maturation, and survival, synaptic transmission, an equilibrium between neuroregeneration and neurodegeneration, as well as memory formation (9). Therefore, there is reason to believe that two proteins—BDNF and IGF-1—can be used as marker molecules for the MDD diagnosis and therapy effectiveness (7, 8).

Recent studies have shown lower BDNF concentrations in the brains of suicidal MDD persons (10, 11). Further, postmortem studies of MDD individuals revealed an association between the diminished BDNF expression in hippocampal areas and a decrease in the volume of these anatomical structures (12). According to meta-analyses, serum and plasma BDNF reductions have been noted in antidepressant-free individuals with MDD as compared to healthy subjects (13–15). The decreased peripheral BDNF levels normalized in reprisal for several interventions [e.g., antidepressants (16, 17), electroconvulsive therapy (18, 19), aerobic exercise (20)]. Thus, we can reasonably assume that the decline in peripheral (plasma/serum) BDNF levels reflects its reduced expression in the brain and could serve as a neurobiological marker of impaired neuroplastic processes in MDD. In addition, the increase of the neurotrophin concentration could indicate therapy effectiveness.

According to our recent systematized data, there are discrepancies in IGF-1 levels in MDD patients across the studies, although the majority demonstrate higher levels of peripheral IGF-1 compared to healthy controls (HC) (8). The elevation of peripheral total IGF-1 concentrations and its decline after antidepressants` treatment were established in several studies in MDD patients (21–23), moreover, it was reported that only patients in remission had attenuated IGF-1 concentrations following treatment (21, 22). The enhancement of the peripheral IGF-1 expression in MDD may be a compensatory mechanism in response to its brain synthesis decrease (24) or diminished neurotrophin bioavailability due to the hyposensitivity of IGF-1 receptors under the neuroinflammatory stress (25). In our recent work, we assumed that the activity of the cerebral-hepatic axis rise in response to inadequate cerebral IGF-1 levels (26). As a consequence, the production of IGF-1 in the liver increases. When cerebral IGF-1 production is restored, the liver IGF-1 production and its blood concentration decrease.

However, the clinical value, sensitivity, specificity, and predictive significance of a single biomarker for MDD remain doubtful. To solve this problem, the approach that uses the cumulative sensitivity and specificity of biomarkers` combination might be applicable (27). Therefore, in our study we aimed: 1) to evaluate if BDNF and IGF-1 serum concentrations separately and in combination might be biomarkers for MDD diagnosis and treatment response; 2) to evaluate the relationships between clinical MDD parameters and serum levels of the above-mentioned neurotrophins.

## Materials and Methods

### Study Design

This was a case-control study, which included 73 participants aged 18 to 65 years. Outpatients (n=41) with MDD diagnosis according to DSM-5 criteria (28) were included through Zaporizhzhia Regional Clinical Psychiatric Hospital, Ukraine. Eligibility criteria for the study participants were described elsewhere (29). Before entering the study, all patients had received no actual antidepressant medication. Subjects were excluded if they had any other psychiatric diagnosis, high suicidal risk, substance dependence/abuse over the past year, significant neurological disorders, head trauma, unstable medical conditions, history of endocrine diseases, psychotic symptoms, the risk for the hypomanic switch (29). Thirty-two healthy controls (HC) with no current psychiatric disorder were enrolled within the same period that the MDD patients were included. HC were excluded based on the use of medications and/or illicit drugs; the intake of alcohol within 48 h of the study visit; and the presence of an unstable medical condition, which could affect cognitive function (29).

All the participants gave written informed consent to take part in the study and attended a baseline visit to undergo the evaluations. The appraisal was reiterated in 30 patients after 8 weeks of vortioxetine treatment 10‑20 mg per day.

The study was approved by the local ethics committee and performed following the ethical standards laid down in the 1964 Declaration of Helsinki and its later amendments and registered at ClinicalTrials.gov (NCT03187093).

### Clinical Assessments

The severity of depression at baseline and changes after treatment were assessed with MADRS (30), Clinical Global Impression Severity (CGI-S) and Improvement (CGI-I) scales (31). Perceived Deficit Questionnaire-5 (PDQ-5), which measures perceived difficulties of cognitive functioning, was used to evaluate subjective cognitive functioning (32). Functioning was rated using the Sheehan Disability Scale (SDS) (33).

### Neuropsychological Assessments

To describe neurocognitive functioning, a battery of neuropsychological tests covering the most impaired cognitive domains in MDD was administered to all participants. The tests were managed using paper and pencil. The instruments included:

1. Rey Auditory Verbal Learning Test (RAVLT), to evaluate immediate verbal memory, retroactive and proactive interference effects, delayed recall, and recognition (34);
2. Trail Making Test B (TMT-B), to assess processing speed, executive function i.e., set-shifting (34);
3. Digit Symbol Substitution Test (DSST), to assess processing speed, executive function, learning, memory, attention, and concentration (34).

The procedures of the tests were described previously (29).

### BDNF and IGF-1 Measurements

In MDD patients and HC, BDNF, and IGF-1 concentrations were evaluated in the morning. Blood was taken by a venous puncture between 8:00 and 11:00 a.m. within the first 2 days after clinical assessments in 73 participants at baseline and 30 patients after 8 weeks of vortioxetine (10‑20 mg per day) treatment. Blood was centrifuged at 3000 g for 10 min, and the serum was stored at -20° C until further processing. IGF-1 was assessed with the chemiluminescence immunoassay Immulite 2500 (Siemens AG, Germany) and Human IGF-1 Quantikine ELISA Kit (R&D Systems Inc., Minneapolis, United States). The measurement range for IGF-1 was from 20 to 1600 ng/ml. BDNF was measured using the chemiluminescence immunoassay «Sunrise» (TEKAN, Austria, GmbH) and BDNF Sandwich ELISA Kit («Millipore-ChemiKineTM»). The measurement range was from 15 to 1000 pg/ml.

### Statistical Analysis

Data analyses were done with the SPSS for Windows, Version 20.0 (SPSS Inc., United States). The results were presented as median (interquartile range) or means (SDs) or percentages. The statistical significance of between-group comparisons was determined using non-parametric and parametric criteria when appropriate. The relationships between demographic and clinical parameters and BDNF or IGF-1 concentrations were assessed with Spearman’s or Pearson’s correlation coefficient. Thereafter, the areas under the receiver operating characteristic curves (AUC-ROC) were calculated to determine if the value of serum BDNF or IGF-1 level could discriminate patients with MDD from HC. A cutoff was derived from the ROC curve with empirical optimal sensitivity and specificity. In addition, a ROC curve for the combination of two biomarkers (BDNF and IGF-1) was built to assess if this combination has a higher value to discriminate patients with MDD from HC. For this purpose, we ran a binary logistic regression to get the probability and built a ROC curve using the probability as the test variable. The statistical threshold was set at p < 0.05.

## Results

### Characteristics of Controls and MDD Patients

**Table 1** demonstrates the main demographic, psychopathological, neuropsychological, and functional characteristics of MDD and HC groups. There were no differences in age, gender, and level of education between groups. Surveyed cohorts had a higher percentage of women than men. The mean years of education in HC and MDD patients was 15 years. Although the number of people with predisposing factors (those that put a person at risk of developing MDD, which included the presence of childhood psychotrauma, persistent stress, and MDD in relatives) was higher in MDD patients, only the presence of precipitating factors (specific events or triggers to the onset of the current DE) significantly differed MDD patients from HC.

**Table 1:** Demographic, psychopathological, neuropsychological, functional characteristics, and serum BDNF and IGF-1 levels in healthy controls and MDD patients.

Variables	HC (n = 32)	MDD (n = 41)	*p*	Treatment group(n = 30)		*p*
				***Pre-treatment***	***Post-treatment***	
***Demographic characteristics***						
Women, n (%)	20 (62.5)^b^	27 (65.9)	0.77	20 (69)	–	
Age, years^§^	38.0 (12.2)^a^	36.4 (12.8)	0.60	35.1 (12.9)	–	
Education, years^§^	15.4 (1.9)^a^	14.7 (2.0)	0.16	14.6 (2.1)	–	
Childhood psychotrauma, n (%)	4 (12.5)^b^	13 (31.7)	0.05	10 (34.5)	–	
Persistent stress, n (%)	12 (37.5)^b^	22 (53.7)	0.17	14 (48.3)	–	
Precipitating factors, n (%)	3 (9.4)^b^	26 (63.4)^**^	**<0.0001**	20 (69.0)^**^		
Number of DE	–	1 (1-2)	–	1 (1-2.5)	–	
MDD in relatives, n (%)	4 (12.5)^b^	12 (29.3)	0.09	10 (34.5)	–	
History of DE, n (%)	–	14 (34.1)	–	11 (37.9)	–	
***Clinical assessments***						
*Psychopathological*						
MADRS total score	2 (0-3.5)^c^	28 (21.5-31.5)^**^	**<0.0001**	29 (24.5-33)^d^	**5 (2.5-10)^##^**	**<0.0001**
CGI-S score	1 (1-1)^c^	4 (4-4.5)^**^	**<0.0001**	4 (4-5)^d^	**2 (1-3)^##^**	**<0.0001**
*Patient-reported cognitive symptoms*						
PDQ-5 total score	1 (0-2)^c^	6 (4-11.5)^**^	**<0.0001**	7 (4-12.5)^d^	**2 (1-4)^##^**	**<0.0001**
*Neuropsychological*						
RAVLT immediate recall total	65.5 (58.25-69)^c^	50.5 (45-54.5)^**^	**<0.0001**	51 (45-54)^d^	**69 (65-72.5)^##^**	**<0.0001**
RAVLT proactive interference	8 (6-9)^c^	6 (5-7)^**^	**<0.0001**	6 (5-7)^d^	**8 (6-9)^##^**	**<0.0001**
RAVLT retroactive interference	15 (13.25-15)^c^	11 (9-13)^**^	**<0.0001**	11 (9-13)^d^	**15 (14-15)^##^**	**<0.0001**
RAVLT delayed recall	15 (13-15)^c^	11 (9-13)^**^	**<0.0001**	11 (9-13)^d^	**15 (14-15)^##^**	**<0.0001**
RAVLT delayed recognition	15 (15-15)^c^	15 (14-15)^*^	**0.003**	15 (13.5-15)^d^	**15 (15-15)^#^**	**0.001**
TMT-B (s) ^§^	52.4 (12.8)^a^	72.3 (21.2)^**^	**<0.0001**	72.4 (23.0)^e^	**45.4 (15.6)^##^**	**<0.0001**
DSST	63 (55.5-68)^c^	50.5 (44.25-59.75)^**^	**<0.0001**	54 (44.5-60.5)^d^	**62 (52-73)^##^**	**<0.0001**
***Patient-rated functioning***						
SDS Work score	0 (0-0)^c^	6 (5-8)^**^	**<0.0001**	6 (5-8)^d^	**1 (0-3)^##^**	**<0.0001**
SDS Social score	0 (0-0)^c^	7 (3.75-9.25)^**^	**<0.0001**	7 (4.5-10)^d^	**1 (0-3)^##^**	**<0.0001**
SDS Family score	0 (0-0)^c^	6 (4-8)^**^	**<0.0001**	6 (4.5-8)^d^	**1 (0-2.5)^##^**	**<0.0001**
SDS Total score	0 (0-1.75)^c^	19 (12-24.5)^**^	**<0.0001**	19.5 (13.25-25)^d^	**2.5 (0-8)^##^**	**<0.0001**
SDS absenteeism, days	0 (0-0)^c^	0 (0-1)^**^	**<0.0001**	0 (0-1)^d^	**0 (0-0)^#^**	**0.007**
SDS presenteism, days	0 (0-0)^c^	3 (2-5)^**^	**<0.0001**	4 (2.25-5.75)^d^	**0 (0-1.5)^##^**	**<0.0001**
***Serum protein levels***						
BDNF (pg/ml)^§^	853.0 (93.9)^a^	727.6 (87.9)^**^	**<0.0001**	737.3 (90.4)^e^	905.3 (59.6)^##^*	**<0.0001**
IGF-1 serum level (ng/ml) ^§^	170.2 (58.2)^a^	289.2 (125.3)^**^	**<0.0001**	288.2 (132.6)^e^	**173.4 (71.2)^##^**	**<0.0001**

Data are presented as median (upper-lower quartile) unless otherwise stated; ^§^data are presented as means (SD).

BDNF, brain-derived neurotrophic factor; CGI-S, clinical global impression—severity of illness; DE, depressive episode; DSST, digit symbol substitution test; HC, healthy controls; IGF-1, insulin-like growth factor; MADRS, Montgomery-Asberg depression rating scale; MDD, patients with major depressive disorder; PDQ-5, perceived deficits questionnaire—5 items; RAVLT, Rey auditory verbal learning test; SDS, Sheehan disability scale; TMT-B, trail making test part B.

a ANOVA (analysis of variance) analysis, controls vs. MDD patients.

b Chi-square test, controls vs. MDD patients.

c Mann–Whitney U-test, controls vs. MDD patients.

d Wilcoxon test (paired samples), “Pre-treatment” vs. “Post-treatment”.

e Paired-samples t-test, “Pre-treatment” vs. “Post-treatment”.

Compared with controls, ^*^p < 0.05, ^**^p < 0.01. Compared with “Pre-treatment”, ^#^p < 0.05, ^##^p < 0.0001.

Besides the expected statistical difference in MDD patients and HC on MADRS and CGI-S total scores, a significant distinction in neuropsychological test performance was found between the comparison groups. MDD participants were significantly worse (p < 0.0001) in executive functioning (DSST, TMT-B scores), processing speed (DSST, TMT-B scores), set-shifting (TMT-B), and all parameters of verbal memory (RAVLT subtests). MDD patients also had a significantly lower level of subjective cognitive functioning as compared to HC.

After 8 weeks of vortioxetine treatment in 30 MDD patients, we revealed that the intake of the antidepressant significantly improved the clinical parameters of patients (**Table 1**). Thus, we observed a significant decrease in depression severity (MADRS, CGI-S, SGI-I scores), improvement of cognitive impairment (measured as subjectively as objectively), and functioning.

### Serum Concentrations of BDNF and IGF-1

**Table 1** shows serum protein levels of BDNF and IGF-1 in HC and MDD patients. It was demonstrated that serum BDNF concentrations were significantly lower in MDD persons compared to HC (p < 0.0001), whereas the concentrations of IGF-1 were significantly higher in patients than HC (p < 0.0001). Although IGF-1 concentrations were slightly higher in women than in men as in MDD [299.4 (131.5) ng/ml vs. 271.5 (116.5)] as in HC group [174.1 (53.4) vs. 164.2 (67.2)], those differences did not reach a significant level.

After 8 weeks of treatment with vortioxetine, BDNF levels were significantly higher in post-treatment than pre-treatment (p < 0.0001), moreover, they were prominently higher than in HC (F = 9.36, p = 0.003). Whereas, IGF-1 concentrations in MDD group post-treatment were significantly lower than pretreatment (p < 0.0001) and not significantly different from HC (F = 1.86, p = 0.18).

### Correlations of Serum BDNF and IGF-1 Levels With Clinical Variables

Next, we performed correlational analyses to determine possible associations between serum BDNF and IGF-1 concentrations and demographic and clinical parameters in the whole sample (**Table 2**). We established prominent inverse relationships between BDNF concentrations and MDD status (r = -0.57, p < 0.01), precipitating factors (r = -0.33, p < 0.01), CGI-S (r = -0.44, p < 0.01), MADRS (r = -0.43, p < 0.01), TMT-B score (r = -0.27, p < 0.05), all subdomains of functioning and positive correlations between serum BDNF levels and RAVLT immediate recall level (r = 0.33, p < 0.01).

**Table 2:** Spearman’s/Pearson’s correlations between demographic, psychopathological, neuropsychological characteristics, and serum BDNF and IGF-1 levels in MDD patients and healthy controls.

Variables	BDNF (pg/ml)	*p*	IGF-1 (ng/ml)	*p*
***Demographic characteristics***				
Age, years	0.02	0.9	-0.17	0.16
Gender	0.15	0.20	0.10	0.44
Depression status	-0.57^**^	**<0.0001**	0.50^**^	**<0.0001**
Persistent stress	0.06	0.59	0.01	0.93
Precipitating factors	-0.33^**^	**0.004**	0.20	0.11
Recurrence of DE	-0.03	0.79	0.16	0.19
Number of DE	-0.04	0.75	0.43^**^	**0.001**
Duration of DE, weeks	-0.02	0.85	0.37^**^	**0.002**
MDD in relatives	-0.12	0.32	0.05	0.68
***Clinical assessments***				
CGI-S score	-0.44^**^	**<0.0001**	0.45^**^	**<0.0001**
PDQ-5 score	-0.19	0.16	0.43^**^	**<0.0001**
MADRS total score	-0.43^**^	**<0.0001**	0.46^**^	**<0.0001**
RAVLT immediate recall total score	0.33^**^	**0.007**	-0.42^**^	**0.001**
RAVLT proactive interference score	0.19	0.11	-0.40^**^	**0.001**
RAVLT retroactive interference score	0.17	0.16	-0.42^**^	**0.001**
RAVLT delayed recall score	0.23	0.06	-0.37^**^	**0.003**
RAVLT delayed recognition score	0.18	0.14	-0.19	0.15
TMT-B (s)	-0.27^*^	**0.03**	0.55^**^	**<0.0001**
DSST score	0.24^*^	**0.048**	-0.45^**^	**<0.0001**
SDS Work score	-0.37^**^	**0.003**	0.40^**^	**0.001**
SDS Social score	-0.46^**^	**<0.0001**	0.39^**^	**0.002**
SDS Family score	-0.38^**^	**0.002**	0.40^**^	**0.001**
SDS Total score	-0.43^**^	**<0.0001**	0.43^**^	**<0.0001**
SDS absenteism, days	-0.12	0.35	0.23	0.07
SDS presenteism, days	-0.36^**^	**0.003**	0.31^*^	**0.01**
***Serum proteins` levels***				
Serum BDNF level	1	–	-0.17	0.18
Serum IGF-1 level	-0.17	0.18	1	–

BDNF, brain-derived neurotrophic factor; CGI-S, clinical global impression—severity of illness; DE, depressive episode; DSST, digit symbol substitution test; HC, healthy controls; IGF-1, insulin-like growth factor; MADRS, Montgomery-Asberg depression rating scale; MDD, patients with major depressive disorder; PDQ-5, perceived deficits questionnaire—5 items; RAVLT, Rey auditory verbal learning test; SDS, Sheehan disability scale; TMT-B, trail making test part B.

*p < 0.05 and **p < 0.01.

As for IGF-1, positive correlations were found between IGF-1 level and MDD status (r = 0.50, p < 0.01), number (r = 0.43, p < 0.01) and duration of DE (r = 0.37, p < 0.01), CGI-S score (r = 0.45, p < 0.01), PDQ-5 score (r = 0.43, p < 0.01), MADRS score (r = 0.46, p < 0.01), TMT-B score (r = 0.55, p < 0.01) and all subdomains of functioning and negative associations between IGF-1 and the performance of RAVLT and DSST tests. Moreover, the correlations between IGF-1 concentrations and performance of cognitive tests were higher than that of BDNF.

### Serum BDNF and IGF-1 Levels for MDD Diagnosis

**Figure 1:**
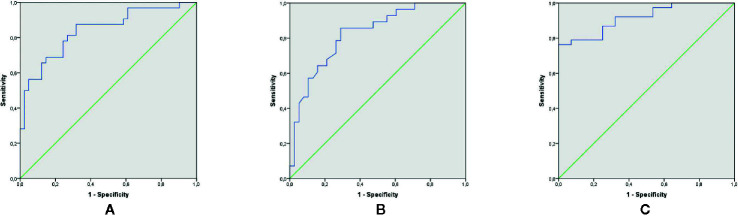
Diagnostic value of serum BDNF and IGF-1 for MDD diagnosis. **(A)** ROC curve for BDNF to diagnose MDD (p < 0.0001, AUC: 0.840; sensitivity; 81% and specificity: 73% for a cutoff of < 763.17 pg/ml). **(B)** ROC curve for IGF-1 to diagnose MDD (p < 0.0001, AUC: 0.824; sensitivity; 84% and specificity: 64% for a cutoff of > 178.00 ng/ml). **(C)** ROC curve the combination of BDNF and IGF-1 to diagnose MDD (p < 0.0001, AUC: 0.916). AUC, Area Under the ROC Curve; BDNF, Brain-Derived Neurotrophic Factor; IGF-1, Insulin-Like Growth Factor; MDD, major depressive disorder; ROC, receiver-operating characteristic.

The discriminating ability of serum proteins’ level to separate MDD participants from HC was determined with ROC analysis. The diagnostic value of BDNF and IGF-1 is shown in **Figure 1**. Separately BDNF (cutoff < 763.17 pg/ml, sensitivity = 81%, specificity = 73%) and IGF-1 (cutoff > 178.00 ng/ml, sensitivity = 84%, specificity = 64%) demonstrated good diagnostic effectiveness, with AUC of 0.840 and 0.824, respectively. However, the combination of two neurotrophins showed excellent diagnostic value for MDD diagnosis, with an AUC of 0.916.

## Discussion

This is the first study to investigate the diagnostic power of the combination of two biomarkers—BDNF and IGF-1—for MDD diagnosis. In our study, we found significantly lower BDNF and higher IGF-1 serum concentrations in patients compared with HC. Following 8 weeks of vortioxetine therapy, serum BDNF levels were significantly higher in post-treatment than pre-treatment and compared to the controls. IGF-1 concentrations in subjects post-treatment were significantly lower than pretreatment and not different from the controls. Significant correlations were obtained between serum BDNF levels and MDD status, the severity of the current DE, presence of precipitating factors, executive functions’ disruption (TMT-B and RAVLT immediate recall scores), and all subdomains of functioning. As for IGF-1, correlations were found between IGF-1 level and MDD status, severity, number, and duration of current DE, subjective and objective cognitive functioning (PDQ-5, RAVLT subtests, TMT-B, DSST scores) and all subdomains of functioning. The associations between IGF-1 concentrations and the performance of cognitive tests were stronger than that of BDNF. Separately both BDNF and IGF-1 demonstrated good discriminating ability for MDD diagnosis; however, the combination of these two proteins had excellent diagnostic power to discriminate MDD patients from HC, providing the AUC of 0.916.

Firstly, we revealed that MDD patients had decreased BDNF concentrations and that these concentrations returned to normal after vortioxetine intake. These results are in line with previous works that found lower serum and plasma BDNF concentrations in depressed persons in comparison with healthy subjects (13, 14, 17, 27, 35–42). Several antidepressants were shown to increase serum/plasma BDNF levels in MDD persons compared to pre-treatment levels, including agomelatine (41), duloxetine (27), escitalopram (27, 43, 44), fluoxetine (41, 45), milnacipran (36), paroxetine (36, 38, 44), sertraline (44), venlafaxine (44), and vortioxetine (17, 46). However, recent works have shown that not all antidepressants increase BDNF concentrations (47), and antidepressant-induced rise in BDNF concentrations are more substantial in responders compared to non-responders (48–51). Moreover, it was demonstrated that high-intensity exercises (52), electroconvulsive therapy (15, 18, 19, 53), and deep brain stimulation (54) also increase BDNF levels. In animal studies, it has been revealed that vortioxetine enhances the behaviors of depressed rodents probably by influencing the cAMP/CREB/BDNF signal pathway and promoting the expression of BDNF in the dorsal and ventral hippocampus of the animals (55, 56). Moreover, it was shown that vortioxetine, but not fluoxetine, raised hippocampal BDNF concentrations in rats (57). Another study demonstrated that vortioxetine amplified the number of synapses and mitochondria substantially, together with increased BDNF levels, while fluoxetine showed no effect (58). It was suggested that fast changes in BDNF concentrations and synaptic/mitochondria plasticity of the hippocampus during vortioxetine treatment may be attributed to vortioxetine’s modulation of 5-HT receptors (58). Longitudinal studies in humans revealed that it is more likely that BDNF is a biomarker for the state of MDD and treatment response rather than a risk factor for MDD (15, 40).

In our study, we obtained significant associations between serum BDNF levels and MDD severity, precipitating factors, executive functions` disruption (performance of TMT-B and RAVLT immediate recall), and all subdomains of functioning. Previous studies in MDD patients have found correlations between BDNF levels and the presence of melancholic features and psychomotor retardation (59), DE severity (36, 60) and duration (60), and cognitive decline during performances of TMT-B (61) and verbal delayed recall (62). Nevertheless, one study evidenced no correlations between patients’ plasma BDNF levels and cognitive functioning (38).

Regarding peripheral IGF-1 increase in MDD patients and its decrease after vortioxetine treatment, our results are consistent with some studies (21, 22, 24, 25, 63–65). Data on a significant decrease of serum IGF-1 concentrations during antidepressant treatment are supported by previous investigations, in which amitriptyline, doxepin, fluoxetine, and paroxetine led to a substantial decline of peripheral IGF-1 levels (21, 22, 66). Nevertheless, one study showed that free IGF-1, not total, concentrations did not change after antidepressant treatment (22). The authors suggested that discrepancies between total and biologically active free IGF-1 levels could be due to adaptation mechanisms to changes typically found in major depression (22).

Although in our study we found no significant differences in IGF-1 levels between women and men in both MDD and HC groups, several previous studies have shown variability across the genders in IGF-1 levels in MDD patients (67–69) These differences between genders may be explained by variations of sex hormone or fluctuations of growth hormone and IGF-1-binding protein (67).

The reported here correlations between IGF-1 level and severity, number, and duration of DE, subjective and objective cognitive functioning (PDQ-5, RAVLT subtests, TMT-B, DSST), all subdomains of functioning are in line with our previous results (23). Nevertheless, here we pointed out that the associations for IGF-1 concentrations and the level of performance of cognitive tests were stronger than those for BDNF levels.

Lastly, the results of ROC-analysis demonstrated that BDNF and IGF-1 separately provided fairly good diagnostic power to separate MDD patients from HC, meanwhile the combination of these neurotrophins showed excellent diagnostic value. Therefore, serum BDNF and IGF-1 levels might be a potential biomarker combination as a diagnostic test for MDD. To the best of our knowledge, we were the first to combine those two serum proteins for MDD diagnosis. Previously it was shown that the arrangement of tPA, BDNF, TrkB, proBDNF, and p75NTR might be a diagnostic biomarker panel for MDD (27) and the combination of BDNF, FGF-2, TNF-α, and 5-HT may predict the efficacy of escitalopram therapy (70).

### Limitations

Our study was limited by its small sample size, therefore, obtained data need to be replicated in larger samples to confirm our findings.

## Conclusions

Patients with MDD had significantly lower BDNF and higher IGF-1 serum concentrations compared to controls. Vortioxetine treatment normalized those disruptions, moreover, after 8 weeks of treatment BDNF concentrations in MDD individuals were significantly higher compared to HC. In the whole sample at baseline BDNF levels correlated with the presence of precipitating factors, meanwhile, IGF-1 levels—with the number and duration of DE. Therefore, we can suggest that BDNF is related to acute stress. As for cognition, IGF-1 had stronger associations than BDNF with the disturbances in different cognitive domains, also, it correlated with the subjective cognitive level. Both factors were associated with functioning in different spheres of life. As BDNF as IGF-1 were significantly associated with MDD status and severity of a current episode and separately demonstrated good discriminating ability for MDD diagnosis. However, their combination had excellent diagnostic power to discriminate MDD patients from HC. Thus, the combination of IGF-1 and BDNF might be considered as a biomarker panel for MDD diagnosis.

## Data Availability Statement

The datasets generated for this study are available on request to the corresponding authors.

## Ethics Statement

The studies involving human participants were reviewed and approved by the local ethics committee of State Institution Zaporizhzhia Medical Academy of Postgraduate Education Ministry of Health of Ukraine. The patients/participants provided their written informed consent to participate in this study.

## Author Contributions

OL supervised the study. OL and AT were involved in the concept of the study and collection of the data. Both authors equally contributed to data preparation and literature search. OL and AT analyzed the data and wrote the first draft of the manuscript. All authors contributed to the article and approved the submitted version. of the manuscript.

## Conflict of Interest

OL is a member of advisory and/or speaker boards of the following companies: Lundbeck, Pfizer, Acino. AT gave presentations for Lundbeck and Acino.

## Abbreviations

AUC-ROC, areas under the receiver operating characteristic curves; BDNF, brain-derived neurotrophic factor; CGI-I, clinical global impression improvement; CGI-S, clinical global impression severity; DE, depressive episode; DSM-5, Diagnostic and Statistical Manual of Mental Disorders, Fifth Edition; DSST, digit symbol substitution test; IGF-1, insulin-like growth factor-1; HC, healthy controls; MADRS, Montgomery-Asberg depression rating scale; MDD, major depressive disorder; PDQ-5, perceived deficit questionnaire-5; RAVLT, Rey auditory verbal learning test; ROC, receiver-operating characteristic; SD, standard deviation; SPSS, statistical package for the social sciences; TMT-B, trail making test B.
